# Retraction: Loss of a Conserved tRNA Anticodon Modification Perturbs Plant Immunity

**DOI:** 10.1371/journal.pgen.1006747

**Published:** 2017-04-24

**Authors:** Vicente Ramírez, Beatriz Gonzalez, Ana López, María José Castelló, María José Gil, Graham J. Etherington, Bo Zheng, Peng Chen, Pablo Vera

The authors of this article [1], with the support of the Instituto de Biología Molecular y Celular de Plantas, are retracting this publication for the following reasons.

The Introduction and Materials and Methods sections of the article contain significant portions of unattributed overlapping text from previously published articles. In the Introduction, the second paragraph contains unattributed text duplicated from the Introduction section in Guy et al., published in 2012 [2], and which summarized distinct aspects of tRNA modifications identified in yeast and humans. Regrettably, while the overlapping paragraph abounds in citations to other authors, the specific citation to Guy et al. (2012) paper was not made. This overlap represents verbatim repetition that was not identified during the submission of the manuscript. In the Materials and Methods, extended text duplications from previous publications of the same authors were identified, in particular from references 6, 13 and 23 and Dobón et al. [3].

The lower panel of Fig. 3D contains images of representative leaves from Arabidopsis plants showing characteristic symptoms of chlorosis due to infection by *Pseudomonas syringae*. Error in selecting the appropriate image folder during the assembling of this figure resulted in some of the images shown in Fig. 3D being incorrectly reused and mislabeled from Fig. 2 in the authors’ previous publication, cited as reference 23. Specifically, images labeled as *Col-0*, *scs9-1* and *npr1* do not represent the genotypes indicated. The scientifically relevant quantitative data shown in the upper graph of Fig. 3D are not affected by this image selection error. The authors maintain that this error does not affect the interpretation of the results nor the conclusion of the study. Although replicates are available for this figure, the authors believe that the preparation of this panel of images, in addition to the instances of overlapping text outlined above, fell below the standard required for publication and therefore the authors and editors agree that the correct action is to retract the article.

The authors apologize to the readers and editors of *PLOS Genetics* and will seek to publish a corrected manuscript version in the future corroborating the findings of this work.

The text of this retraction notice has been agreed to by all authors and the Editors-in-Chief.
